# Aphid-Resistant Plant Secondary Metabolites: Types, Insecticidal Mechanisms, and Prospects for Utilization

**DOI:** 10.3390/plants13162332

**Published:** 2024-08-21

**Authors:** Muhammad Farhan, Jilong Pan, Hammad Hussain, Jun Zhao, Hanjing Yang, Ishtiaq Ahmad, Shuai Zhang

**Affiliations:** 1 College of Plant Protection, Yangzhou University, Yangzhou 225009, China; farhan.entomology@gmail.com (M.F.); mz120231454@stu.yzu.edu.cn (J.P.); 19906139606@163.com (J.Z.); yanghj926445@163.com (H.Y.); 2 College of Horticulture and Landscape Architecture, Yangzhou University, Yangzhou 225009, China; hussainhammad788@gmail.com; 3 Department of Horticultural Sciences, The Islamia University of Bahawalpur, Bahawalpur 63100, Pakistan; ishtiaq@iub.edu.pk

**Keywords:** plant secondary metabolites, aphids, aphid damage, insect-resistant PSMs, herbivore-induced volatiles, biopesticides

## Abstract

Aphids pose a significant threat to global agricultural crop production, leading to widespread pesticide use and resistance. This necessitates the use of alternative substances, like plant secondary metabolites (PSMs). Plants have developed protective compounds known as alkaloids, terpenoids, phenolics, sulfur- and nitrogen-containing metabolites. These compounds exhibit promising characteristics against aphids, such as antifeedant, aphicidal, and disrupting survival fitness. This review highlights the importance and application of secondary metabolites in combating aphid populations. Different insect-resistant substances have different mechanisms for managing aphids and other pests, including defensive signaling, inhibiting growth, and attracting natural predators by releasing herbivore-induced volatiles (HIPV). The application of plant secondary metabolites as biopesticides has proven to be an effective, economical, and eco-friendly alternative to synthetic pesticide chemicals. Furthermore, this review comprehensively discusses the principle role of plant secondary metabolites, encouraging sustainable agricultural practices and emphasizing the integrated management of the aphid population.

## 1. Introduction

Aphids (Aphididae, Hemiptera) are significant crop pests across a substantial portion of the world, causing considerable economic harm by eating directly on crops, excreting honeydew, and transmitting viruses [1]. The economic losses caused by aphids are substantial, with millions of dollars being lost annually worldwide [2]. This underscores the urgent need for effective pest management strategies. Aphids have more than 5000 species throughout the world [3]; out of these, approximately 450 species have been documented from crop plants, whereas 100 species have been observed as important agricultural pests causing significant economic losses to the crops [4]. Aphid attacks become more severe with the carbon dioxide concentration in the atmosphere and nitrogen application in fertilizer form [5]. Aphids can reproduce in both sexual and asexual ways depending on the environmental conditions [6]. Aphids have the capacity for rapid evolutionary changes due to parthenogenesis and polyvoltinism. These changes are characterized by frequent breeding, polyphagy, and a high degree of polymorphism. Aphids also exhibit anholocyclic/holocyclic reproduction and host alternation [7]. Aphids in temperate zones display holocyclicity, which involves the production of both male progeny and oviparae. The oviparae then mate to generate eggs that will hibernate [8]. Hardie [9] investigated the influence of nutrition on the development of morphs and offered a comprehensive life cycle pattern of aphids. The wingless viviparous female, known as “the multiplier”, quickly begins parthenogenesis, resulting in a fast population increase. As winter approaches, most wingless viviparous insects change into wingless egg-laying females, while winged viviparous insects develop into winged males towards the end of the year. After engaging in sexual reproduction, fully developed female organisms return to their main hosts to lay fertilized eggs [10].

Due to their rapid and alternative ways of development, it is not easy to control them under the economic threshold level. However, the most effective and easy method of controlling aphid population in crop fields is the application of insecticide chemicals. However, these insecticides are no longer effective and desirable because of rapid resistance development [11], negative impacts on the non-target organisms, and the desire for low insecticide influence [12], to promote integrated pest management (IPM) strategies. The widespread insecticide resistance is threatening the productivity of crops. However, plant extracts have evolved as alternative biopesticides for effective and environmentally friendly pest control [13,14]. Additionally, these biopesticides can effectively reduce the excessive use of insecticides and give ideas for novel biopesticides development [15]. In this regard, plant secondary metabolites have evolved as resistant compounds against different agricultural herbivores. However, where the primary metabolites are of great importance, these secondary metabolites are regarded as unimportant for other developmental processes of plants [16]. In plant biomass, primary metabolites are involved in plants’ normal development and growth, whereas secondary metabolites are the compounds engaged in the defensive mechanism against different herbivores. Though primary metabolites’ function differs from secondary metabolites, they are interconnected because they are the building blocks for secondary metabolite synthesis [17]. Recent studies have reported that PSMs adversely affect the survival fitness of *Myzus persicae* [18] and *Aphis gossypii* [19] after the application of salicylic acid and cucurbitacin B, respectively. These secondary metabolites are eco-friendly and cost-effective compared to other chemical pesticides [20].

This review highlights the latest information regarding PSM’s defensive function against different aphids. We also present different types of plant-resistant secondary metabolites, their role in maintaining aphids’ population, and the mechanism of these compounds to control aphids. This strategy not only uses natural plant defenses but also helps to reduce the need for chemical pesticides, improving ecological balance and environmental health.

## 2. Occurrence and Harm of Aphids on Crop Plants

Aphids are globally distributed especially in temperate zones like sub-Saharan Africa and North America, comprising 1416 aphid species, nearly 1500 species in Europe, and 1000 species in China. In contrast, Australia and South Asia have very low aphid species [21]. In this review, we discuss some species of aphids infesting six important as well as other agricultural crops worldwide: *Aphis gossypii* (cotton or melon aphid), *Lipaphis erysimi pseudobrassicae* (mustard or turnip aphid), *Myzus persicae *(green peach aphid), *Sitobion avenae* (wheat aphid), *Acyrthosiphon pisum (pea aphid)*, *Rhopalosiphum maidis* (corn leaf aphid), *Rhopalosiphum rufiabdominalis *(rice root aphid), and *Aphis glycines *(soybean aphid) (Table 1).

### 2.1. Aphis gossypii and Acyrthosiphon gossypii

*Aphis gossypii* and *Acyrthosiphon gossypii* are the two species of aphids infesting cotton plants; however, *A. gossypii* is regarded as one of the most dangerous polyphagous species, infesting approximately 100 species of agricultural crops worldwide [33]. It also infests many other crops with cotton, like citrus, potato, cucurbits, ornamental plants, peppers, and okra. *Acyrthosiphon gossypii* is infesting legumes and cotton crops and is found in areas like Asia, Europe, and Ukraine [23]. *A. gossypii* is also known as melon or cotton aphid and is predominantly distributed in temperate and tropical regions throughout the globe. It effectively transmits over 50 plant viruses and is regarded as a major economic and destructive pest worldwide [34]. It mainly infests buds and, underneath the leaves, sucks plant sap, posing significant damage resulting in retarded plant growth, malformed buds, and withered leaves [35]. After sucking the plant sap, aphids excrete honeydew that promotes the growth of sooty mold on the leaves. As a result, plants’ respirations and photosynthetic capacity decrease and become weak. If not controlled, *A. gossypii* can reduce up to 44% production by transmitting different plant viruses [36].

### 2.2. Lipaphis erysimi pseudobrassicae

*Lipaphis erysimi pseudobrassicae* is commonly known as a mustard or turnip aphid, posing more than 50% loss in production, and is considered the destructive brassica pest. Likewise, *A. gossypii* also has the same feeding behavior of sap-sucking from the plants, excreting honeydew, inhibiting plant growth, and developing sooty mold. Additionally, *L. erysimi* transmits more than 13 types of viruses in the *Brassicaceae* family, including *Turnip mosaic*, *Radish mosaic*, *Cauliflower mosaic*, and *Beet mosaic* virus [24]. This mustard aphid is widely occurring in Asia, is also a destructive pest of cruciferous crop plants, and is sometimes difficult to control [37]. Serious infestations of turnip aphid have been reported in different countries of Africa, including South Africa, Benin, Sudan, Egypt, and Mali [25].

### 2.3. Myzus persicae

*Myzus persicae*, the green peach aphid, is of great agricultural importance, infesting over 800 plant species worldwide [38]. *Myzus persicae* is also known as potato aphid because it also feeds on potato crop plants and causes heavy economic damage [39]. It feeds on plant sap, causes necrosis and chlorosis spots, thereby reducing the market value of fruits, and effectively transmits persistent and non-persistent viruses. It is reported that this aphid originated in China and is now widely distributed in China and Australia, whereas a very low population occurred in Asia and Europe [26].

### 2.4. Sitobion avenae

*Sitobion avenae* and *Schizaphis graminum* are the two main cosmopolitan aphid species infesting wheat crops. *Sitobion avenae* seriously threatens wheat production in China, reducing 40% yield by infesting over 13 million hectares annually [27]. *Sitobion avenae* is also known as a grain aphid, predominantly infesting wheat crops in Northern China, some regions of the Yangtze River, West Africa, Europe, and some Asian countries [40].

### 2.5. Acyrthosiphon pisum

Furthermore, *Acyrthosiphon pisum*, commonly known as the pea aphid, is also a cosmopolitan and destructive pest of many legume crop plants [41]. Pea aphids can transmit up to 30 viruses, including red clover vein mosaic, pea streak, and yellow mosaic virus [42]. As a result, significant economic losses of USD 60 million have been reported in the USA annually, with a 10 to 30% loss in Northwest China. Symptoms of pea aphids feeding on pea plants include retarded growth, curling of leaves, and wilting of plants [28].

### 2.6. Rhopalosiphum padi

Some species of aphids have different modes of infestation and feeding behavior, such as at the time of early development they feed on upper parts of the plants. Similarly, the damage and economic loss caused by aphids varies with host plants and aphid species. In this regard, the grain aphid is considered the most destructive pest, causing serious yield losses by infesting spikes and grains as compared to *Rhopalosiphum padi*, commonly called bird cherry oat aphid, which does not cause serious symptoms on the host plants but reduces plant growth when population levels increase. *Rhopalosiphum padi* also damages maize crops by sucking plant sap [40].

### 2.7. Rhopalosiphum maidis

*Rhopalosiphum maidis* is known as a corn leaf aphid, which predominantly attacks two important cereal crops including maize and barley worldwide [43]. It also attacks other monocot plant crops like sorghum, wheat, and oats. Its nymphs and adults cause subsequent damage by sucking plant sap from the phloem, curling of leaves, yellowing and drying of leaves, and transmitting different viruses, thereby posing significant yield losses from 17.1 to 100% in India in Barley crops [29]. *Rhopalosiphum maidis* and *Rhopalosiphum padi* are cosmopolitan pest species of aphids and occur widely worldwide, including in China, America, Europe, New Zealand, Egypt, Canada, North Africa, Australia, and India. These aphids transmit different viruses in maize and barley crop plants like maize dwarf mosaic and barley yellow dwarf virus [30].

### 2.8. Rhopalosiphum rufiabdominalis

*Rhopalosiphum rufiabdominalis* is a rice root aphid that feeds on the roots of rice plants and makes a defensive white wax that can absorb extra moisture. As with other aphid species, they are also produced asexually using parthenogenesis. The winged aphids mostly live above the ground and move to other plants, while the wingless aphids live below ground and feed on the roots of rice by sucking phloem sap [44]. This aphid species is also cosmopolitan, found in Asia, and widely occurs in the USA, where it was reported for the first time in the roots of the cotton plant. Moreover, this species is also found in the roots of other plant species like grasses, tomatoes, and wheat [31].

### 2.9. Aphis glycines

Similarly, *Aphis glycines*, also known as soybean aphid, is found in East Asia but predominantly distributed in nearly 30 states of the USA where it was first reported in 2000. Soybean aphids caused up to 40% yield loss in Minnesota by sucking plant sap and reducing the quality of seed. Like other aphid species, soybean aphids also transmit deadly plant viruses, including soybean mosaic and alfalfa mosaic virus, and secrete honeydew which promotes fungal growth and reduction in photosynthetic activity, resulting in serious economic damages [32].

## 3. Types of Plants-Insect Resistant Substance

**Figure 1:**
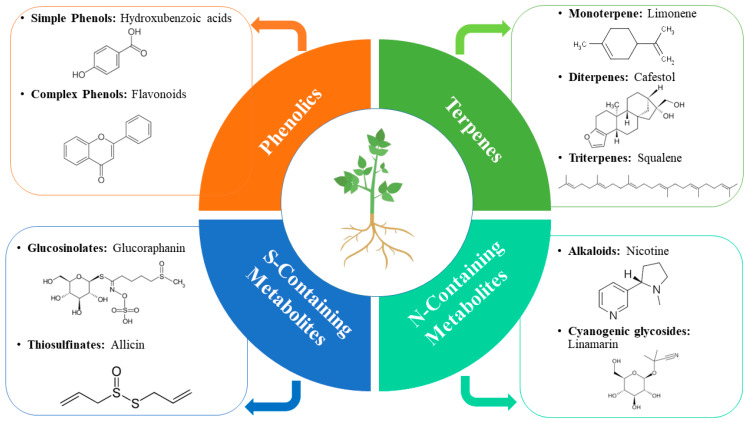
Types of PSMs with some chemical structure examples.

Plants have evolved different insect-resistant substances for defensive mechanisms. These substances include physical barriers like trichomes, plant volatile organic compounds biosubstances, vegetal substances, biomolecules, and plant secondary metabolites. Here, we focused on plant secondary metabolites. Plants use two different defensive ways to resist insect attacks. In the first case, they emit specific volatile chemicals that repel the insects and resist oviposition. However, in the second case, in response to insects feeding on plant tissues, they produce different antibiotic substances like secondary metabolites, which increase the mortality of insects. PSMs have emerged as potential defensive chemical compounds against herbivores. Plants also use secondary metabolites to resist microorganisms’ infection. There have been over 2,140,000 secondary metabolites discovered from plants so far [45]. Even though there are many types of plant compounds which are found to be bioactive against aphids, the PSMs are mostly divided into four distinct groups: phenolics, terpenoids, sulfur- and nitrogen-containing compounds (Figure 1).

### 3.1. Phenolics

Phenolic compounds are a diverse group of PSMs comprising over 10,000 toxic compounds. These compounds showed toxic and repellent activity against different insect herbivores and pathogens [46]. These extensive groups of chemical compounds are essential for plant survival and development because of their important biochemical, physiological, and defensive properties against pests in different biotic and abiotic processes. Phenolics are usually categorized into simple (phenolic acids, hydroxybenzoic acids, and catechols) and complex (lignins, tannins like tannic acid, flavonoids, etc.) phenols comprising a phenyl group (hydroxyl) with an aromatic ring of benzene [47]. Phenols are produced in plants in response to herbivores feeding or pathogen infection using a specific pathway called the phenylpropanoid pathway. This is an important phenolic pathway in plants that the corresponding genes activate due to insect infestation or pathogen infection [48].

### 3.2. Terpenes

Terpenes, commonly called terpenoids, are considered the principal and diverse group of PSMs comprising 25,000 chemical compounds [49]. Studies revealed that these compounds are found in approximately all plants and play an important role in plant defense against insect pests [50]. Commonly, terpenes are produced by isomers of isopentyl diphosphate (IDP) and dimethylallyl diphosphate (DMAPP); however, units of 5-C isopentane play an important role in their formation [49]. Likewise, phenolic compounds and some terpenes, including brassinosteroids and gibberellins, are also served in plant growth and developmental processes. Terpenes are also involved in insect pest feeding and oviposition repellence. It has been observed that some insect herbivores can carry these compounds while feeding on the plants and use them as stimulants in locating the corresponding host plant. Moreover, terpenes are essential in attracting beneficial insects and crop pollinators by releasing volatile compounds [51].

### 3.3. Sulfur-Containing Plant Secondary Metabolites

Sulfur-containing PSMs are demonstrated as glucosides amino acid derivatives with nearly 120 molecular structures, especially in *Capparales* and *Brassicaceae* plants [52]. Mostly, sulfur-containing chemical compounds are volatile, have an unpleasant smell, and are bitter in taste. Many sulfur-containing PSMs have been reported, including thiosulfinates, glucosinolates like allicin derived from cysteine sulfoxides, and antimicrobial peptides like thionins and defensins [53]. These chemical compounds are categorized into two main groups based on their formulation pathways. The first group comprises glucosinolate, which is formulated by myrosinase enzyme hydrolysis. *Crucifereae* family plants exhibit this specific pathway, including broccoli, nasturtium, and cabbage. It has been observed that glucosinolate is mostly found in plants’ reproductive parts and young leaves. However, the second group includes alliin, mainly present in plants associated with the genus Allium, such as onion, garlic, and leeks. Alliin is formed as a result of alliinase enzyme hydrolysis. These two pathways help plants defend against herbivores and pathogenic infections [50].

### 3.4. Nitrogen-Containing Plant Secondary Metabolites

Alkaloids, cyanogenic glycosides, and some essential plant growth hormones like auxin are the commonly known nitrogen-containing PSMs [45]. Nearly 10,000 alkaloids have been discovered so far [49] from angiosperms, gymnosperms, and other plant genera [54]. Alkaloids are the main class of nitrogen-containing PSMs and are toxic to insects because of their distinct mode of action. These chemical compounds directly affect the insect nervous system, disturb the replication of DNA, and ultimately interfere with enzyme function and production of proteins [55]. Nitrogen constitutes 2% of total plant biomass; therefore, these compounds are found in plants in high amounts. Moreover, alkaloids are classified into three distinct groups including true, pseudo, and proto alkaloids. True alkaloids originate from amino acids with a precursor of N-heterocyclic ring, mainly including morphine, atropine, nicotine, and quinine. Naturally, true alkaloids are crystalline and form salts soluble in water, thereby considered highly reactive chemical compounds. Nicotine is the only true alkaloid that occurs in brown liquid form. However, all other types of true alkaloids are found in solid form and have a bitter taste. Unlike true alkaloids, pseudo alkaloids are not derived from amino acids, including caffeine, capsaicin, or solanidine [56]. Likewise, true alkaloids and proto alkaloids are also the derivatives of amino acids that are structurally simple such as hordenine, yohimbine, and mescaline. Interestingly, the nitrogen atom in proto alkaloids does not take part in the heterocyclic ring derived from amino acids [57].

## 4. Application of Insect-Resistant Substances to Control Aphids

Aphids are the important agricultural economic pests posing huge losses to crops worldwide. The application of pesticide chemicals is the main way of managing the aphid population below the economic threshold level. Excessive use of these chemicals creates many problems, such as producing insect resistance, which is the main disadvantage, polluting the environment, disturbing the food chain, and posing a drastic impact on beneficial insects and crop pollinators. In this regard, there is a crucial need to utilize insect-resistant substances to overcome these problems. Plants’ secondary metabolites are considered eco-friendly and beneficial toxic substances for managing aphid populations. Biopesticides development and application are important to understand the impact of PSMs against aphids [19]. The literature revealed that PSMs have been successfully utilized as biopesticides against aphids and other insect species. For example, nicotine extracted from tobacco plants is a secondary metabolite used against different herbivore insects because of its repellent activity [58]. In this regard, tobacco plants stop the production of nicotine chemical compounds on *Manduca sexta* (Tobacco hornworm) infestation and start emitting E-bergamotene. This volatile compound helps in the attraction of *Manduca sexta* predators. Similarly, triterpene azadirachtin and pyrethrin are regarded as two biopesticides and important secondary metabolites because they have minimal toxic effects on non-target living organisms [59].

Many terpenoid-based biopesticides have been reported, including citrus oils, neem extracts, and oil extracted from *Chenopodium ambrosioides* which comprises 14 monoterpenes. The d-limonene is a terpenoid chemical compound classified as monoterpene extracted from citrus plants and has a toxic effect against different insect herbivores [60,61], including *Aphis gossypii* and *Myzus persicae* [62]. Azadirachtin, known as triterpene, derived from the neem plant, possesses repellent and toxic properties against peach aphids [63]. Similarly, in the past, Smith et al. [62] compared the toxic effect of three terpenoid-based biopesticides derived from orange (d-limonene), neem (azadirachtin), and *Chenopodium ambrosioides* extract with two synthetic pesticides, flonicamid and spirotetramat, against *Myzus persicae* and *Aphis gossypii*. These terpenoid-based biopesticides demonstrated similar toxicity as synthetic insecticides against both aphid species. Farnesol and geraniol are classified as sesquiterpenoid and monoterpenoid PSM, in that order. These PSMs are extracted from lemon grass, citronella rose, and other plants with antifeeding properties against insect pests. These two chemical compounds were employed as biopesticides in past research against *Myzus persicae* and showed more than 90 and 50% mortality [64]. Farnesol and terpenoids also can potentially repel and show toxicity against *Aphis fabae* [65,66]. Tomato plants produce monoterpenes and sesquiterpenes accumulated in glandular trichomes [67]. Recent research on the behavior of *Macrosiphum euphorbiae* (potato aphid) revealed that these terpenes seriously affected feeding behavior, survival rate, and fecundity [68]. Similarly, different types of nine monoterpenes, including bornyl acetate, carvacrol, citronellyl acetate, eugenol, geramyl acetate, linalyl acetate, neryl acetate, terpinolene, and terpinyl acetate, were tested against a cosmopolitan pest *Myzus persicae* and exhibited encouraging toxicity. Furthermore, all these secondary metabolites exhibited the least toxic effect on the non-target Pallas larvae (*Harmonia axyridis*), emphasizing the proper use of PSMs as biopesticides for pest control [69]. In addition to *Myzus persicae* and *Aphis fabae*, further investigation and research are needed to assess these PSMs’ toxicity against other aphid species.

Phenolics are also commonly present in plants after terpenes and play a vital role in plant defense, including flavonoids, caffeic acid, protocatechuic acid, chlorogenic acid, and *p*-cumaric acid, and exhibit a negative impact on crop pests [70,71,72]. The study reveals that phenolic compounds like quercetin negatively affect the survival and fecundity rate against aphids, whereas coumarin and thymol showed a positive impact [73]. Additionally, ferulic acid and *p*-coumaric acid, known as phenolic acids, also have antifeeding properties for insect pests [74]. Many studies revealed that PSMs are involved in trophic-level interactions [75]. The chemistry of host–plant interaction shows a positive response to the attraction of natural predators. For example, some phenolic compounds like coumaric acid and syringic acid have negative impacts on pests *Spodoptera litura* and *Achaea jantata*; however, they boost the parasitic ability of *Trichogramma chilonnis* [76]. The concentration of PSMs in plants increased in response to aphids’ attack. Florenico-Ortiz et al. [77] studied the phenolic composition of pepper leaves after infestation of *Myzus persicae*. Outcomes showed that different phenolic acids, including caffeic acid, sinapic acid, coumaric acid, and cinnamic acid concentrations, were increased in response to the high density of peach aphids. Condensed tannins, classified as polyphenolics present in aspen plants (*Populus termula*), can potentially resist pathogens and chewing pests and can deter aphids [78]. Salicylic acid also has defensive properties against both sucking and chewing types of herbivores as well as pathogens. Research indicates that salicylic acid significantly reduces the fecundity and apterous rate of *Sitobion avenae* (wheat aphid) [79]. Genistein, apigenin, kaempferol, and daidzein are grouped as flavonoids (polyphenolics). These compounds affected the feeding behavior of pea aphid *Acyrthosiphon pisum* and deterred the penetration of stylet [80]. Phenolic acids derived from the leaves of walnut, black current, and sour cherry, including tannic acid, ferulic acid, vanillic acid, *p*-coumaric acid, and chlorogenic acid, significantly prolonged the survival rate and reduced fecundity rate of *Sitobion avenae* (grain aphid) [70]. Epigallocatechin Gallate is one of the most important phenolic compounds found in cotton and green tea plants, potentially decreasing the population level and badly impacting the non-host adaptability of *Aphis gossypii* [19].

Glucosinolates are classified as sulfur-containing plant secondary metabolites derived from Brassicaceae family plants. These compounds varied in concentration in response to insect infestation. Glucosinolates affected the survival fitness of *Myzus persicae* but also reduced the performance of the predators of aphid species [75]. Similarly, sinigrin is a type of glucosinolate that negatively affects the apterous aphid population [81]. The green peach aphid is a serious economic pest; its feeding behavior varies based on host plant nutrients and secondary metabolites concentration. A recent study investigated its feeding performance on different Brassicaceae plants (Chinese cabbage, cabbage, and radish), and the amount of glucosinolates and amino acids was also measured. Aphid preferred to feed on Chinese cabbage compared to radish and cabbage due to the lower concentration of sulfur-containing PSMs glucosinolates and high content of amino acids in leaves. Aphid showed poor feeding behavior and growth in response to higher concentrations of glucosinolates in cabbage and radish [82]. Allium plant species are a rich source of organosulfur chemical compounds like thiosulfinates. Thiosulfinates seriously decrease the survival rate and population rate of *Aphis gossypii* [83].

Alkaloids are the main group of N-containing PSMs with distinct insecticidal properties against aphids and other plant herbivores. Different kinds of alkaloids were extracted from the *Sophora alopecuroides* L. plant, including oxymatrine, cytisine, sophoridine, aloperine, matrine, and sophocarpine. Researchers tested their aphicidal activity against *Myzus persicae*, *Sitobion avenae*, and *Aphis craccivora*. The outcomes showed that all the tested compounds exhibited promising insecticidal activities [84]. Alkaloids also show toxic effects against other insect species, including *Leucania separate*, *Brontispa Longissima*, *Plutella xylostella*, *Plagiodera versicoloraetc*, *Helicovera armigera*, and *Aphis laburnis* [85]. Different aphid species have different feeding behaviors and host preferences in response to PSM concentration. The host plants with low content of defensive chemical compounds are susceptible to insect pest attack compared to those with high concentrations of PSMs. Breeding of plants developed such plants, including *Lupinus angustifolius* (Narrow-leafed lupins), with less PSM content, which increased the susceptibility to aphid attack. The study was conducted to assess the feeding habits of different aphid species, including *Aphis craccivora*, *Myzus persicae*, *Aphis fabae*, *Macrosiphum albifrons*, and *Acyrthosiphon pisum* on *L*. *angustifolius* plants with different concentrations of alkaloid for a 12 h period. The results demonstrated that the probing time of *A. pisum*, *A. fabae*, *M. persicae*, and *A. craccivora* increased on reduction in alkaloid concentration. However, *M. albifrons* showed no influence [86]. Furthermore, Park et al. [87] extracted various alkaloids from *Corydalis turtschaninovii* tuber, including corydaline, isocorypalmine, pseudoprotopine, glaucine, demethylcorydalmine, and stylopine, and tested their aphicidal activity against *Aphis gossypii*. The observed outcomes revealed that these PSMs indicated promising insecticidal activity. The literature review describes that PSMs have significantly principle defensive, antifeeding, and aphicidal properties. This positive response promotes and provides better emphasized and integrated strategies for properly applying PSMs as biopesticides at the economic level instead of synthetic ones. In the following table, we allocated different PSM functions against different aphid species (Table 2).

## 5. Mechanisms of Insect-Resistant Substances to Control Aphids

**Figure 2:**
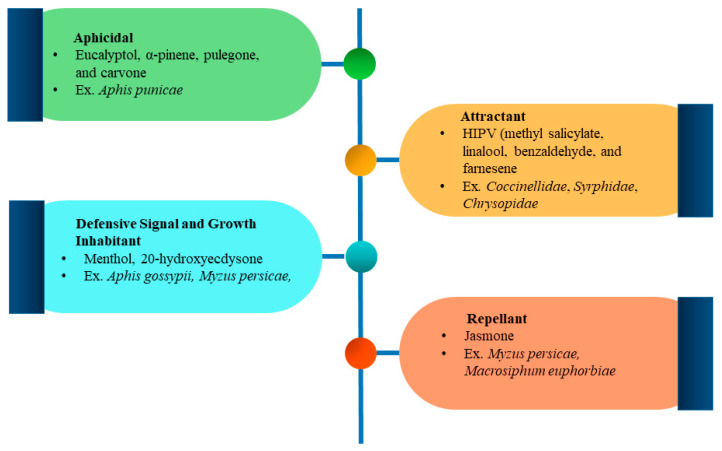
Direct and indirect defense mechanism of insect-resistant PSMs to control aphids, with examples.

Plants have evolved a variety of biochemical compounds to protect themselves from biotic and abiotic factors that harm their growth, development, and survival. These chemical compounds are regarded as primary and secondary metabolites. The primary ones regulate growth and development, whereas secondary metabolites are responsible for defensive mechanisms. PSMs perform defensive mechanisms in direct and indirect ways against biotic factors. Direct defense includes herbivores attacks, including aphicidal and repellant activity, growth inhibition, and defensive signaling; however, indirect defense involves attracting natural enemies of herbivore pests (Figure 2).

### 5.1. Direct Defense

#### 5.1.1. Aphicidal and Repellant Activity

Plants’ secondary metabolites are likely categorized into four major groups: terpenes, phenolics, sulfur- and nitrogen-containing compounds. These compounds are directly or indirectly involved in deterring or killing insect herbivores. Antifeedant is a property of a particular secondary metabolite compound that does not involve killing the attacking insect but deters it from chewing or sucking plant material. The attacking insect herbivore remains stuck in the parts of plants without eating until death. These compounds have been proven to be eco-friendly and defensive biochemical substances. As far as the mechanism of antifeedant activity in herbivores is concerned, it is still unclear; however, it could be considered that antifeedant substances decrease or stop eating because of an unpleasant taste or smell [136]. For example, azadirone, salannin, azadirachtin 7-deacetyl, methyl angolensate, gedunin, and azadiradione are classified as triterpenoids derived from *Meliaceae* reported as potential antifeedants against numerous insects, especially lepidopterans [137]. Furthermore, some limonoids (triterpenoids) like dumsenin, dumnin, musiduol, and musidunin derived from *Croton jatrophoides* are also regarded as antifeedant against different insects [138]. Pyrethrins are the most important PSMs and show similar deterring properties against some sucking insects like aphids *Myzus persicae* [139]. PSMs are the main constituents of essential oils extracted from plants and can be used as antifeedant, repellant, or aphicidal. These plant extracts demonstrated promising antifeedant, aphicidal, toxic impact, or deterrent properties against pests. For example, essential oils extracted from *Salvia rosmarinus*, *Salvia officinalis*, *Mentha longifolia*, and *Mentha piperita* contained secondary metabolites, including α-pinene, eucalyptol, pulegone, and carvone, respectively. These plants’ chemical compounds showed principal repellent, antifeedant, and aphicidal properties against *Aphis punicae* [140]. Similarly, jasmone is a sesquiterpenoid, and has potential aphid-repellent activity against two aphid species, including *Myzus persicae* and *Macrosiphum euphorbiae* obtained from *Jasminum* spp. and *Camellia sinensis* [141].

#### 5.1.2. Defensive Signaling and Growth Inhibitor

Plants utilize some types of secondary metabolites such as terpenes, aromatics, benzoxazinoids, volatile compounds emitted from green leaves, glucosinolates, and cyanogenic glycosides as potential defensive signals or regulators and against herbivores [142]. Hydrogen cyanide is a cyanogenic glycoside that inhibits the binding of oxygen atoms to cytochrome-c and causes respiration disturbance, leading to the death of insects. For example, dhurrin is also a cyanogenic glycoside emitted from *Sorghum bicolor*, inhibiting the feeding of *Chilo partellus* larvae and *Acrida exaltata* [143]. Some plant probiotics, like the bacteria *Pseudomonas chlororaphis*, emit hydrogen cyanide that inhibits insect herbivore growth, like *Myzus persicae* [144]. Similarly, some monoterpenes named isomenthone, menthol, and neomenthol originated from *Mentha piperita*, and *Mentha arvensis* lowered the fecundity rate of *Aphis gossypii* [97]. PSMs indirectly disturb the insect developmental process. For example, ecdysteroid hormone is involved in insect development, which is disturbed by the naturally occurring analogue of plants, 20-hydroxyecdysone (Phytoecdysteroids) [145]. These secondary compounds are structurally similar to insect growth or molting hormone ecdysone by mimicking. These chemical compounds naturally occur in nearly 6% of plants, such as *Asparagus* spp., *Spinacia oleracea*, and *Chenopodium quinoa* [146]. The study revealed that the 20-hydroxyecdysone compound reduced the development and growth rate of aphid *Myzus persicae* [147]. Saponins are glycoside triterpenoids that showed effective toxicity against *Acyrthosiphon pisum* on ingesting by disrupting the midgut epithelium [93].

### 5.2. Indirect Defense

#### Attractant

**Figure 3:**
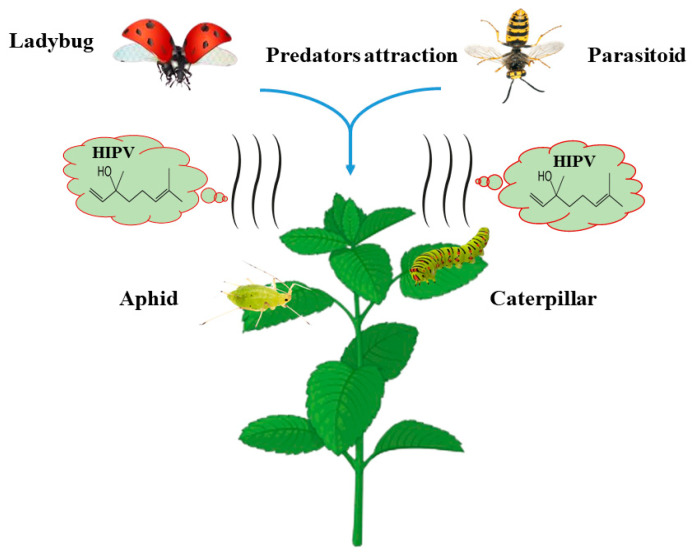
HIPV mechanism of natural enemies attraction.

Plants release some kinds of volatile compounds in response to herbivore attacks called herbivore-induced plant volatiles (HIPV). HIPV emitted in response to insect pests’ attacks attracts their natural predators. These volatile compounds mostly include phenylpropanoids, fatty acids (volatile), amino acids, terpenoids, and benzenoids [148]. Natural predators use these volatile compounds as cues to trace the infested plants and, hence, the respective prey (Figure 3). However, the mechanism of HIPV defense to attract natural predators is unknown, and which type of volatile compound is responsible for drawing specific predators is unclear. However, potato and cucumber plants emit special HIPV chemicals to attract ants in response to aphid and caterpillar infestation [149]. Furthermore, when damaged by caterpillars and aphids, *Arabidopsis* plants release similar volatile chemicals to attract natural enemies like parasitoids *Diadegma semiclausum* [150]. *Trichogramma* wasps are attracted by the volatile compounds released by *Brassica* plants [151]. Similarly, a study was conducted in apple orchards to artificially attract the natural predators of aphids using some HIPV compounds, including methyl salicylate, linalool, benzaldehyde, and farnesene. The study demonstrated that these compounds effectively attracted the *Coccinellidae*, *Syrphidae*, and *Chrysopidae* families’ predators of aphids in apple orchards [152]. Likewise, another study indicated that *Hippodamia variegata* (ladybird), an important predator of *Aphis gossypii*, is drawn by different types of HIPV emitted by plants during aphid infestation [153].

## 6. Prospects

Aphids are causing serious economic losses and a dangerous threat to agricultural crop production. Annually, the world is facing huge crop damage. The occurrence of harm to different types of aphid species suggests using naturally occurring biochemical substances in plants called secondary metabolites. Secondary metabolites are regarded as the natural defensive army against different herbivores and pathogens. Currently, PSMs have become a great interest of study due to their multiple and broad-spectrum importance in maintaining plant health by protecting from herbivores. These chemical compounds are categorized into four groups: phenolics, terpenes, sulfur- and nitrogen-containing metabolites, which demonstrate promising features, including toxicity, antifeedant, probing resistance, low fecundity, and disrupting the survival of aphids. These insect-resistant substances show similar effectiveness in combating different aphid species like synthetic insecticides. PSMs-based biopesticides are eco-friendly and reported no harmful effect on non-target natural enemies or other organisms. Moreover, advances in metabolomics have improved our understanding of PSMs at the molecular level. This approach has shifted our focus from plant breeding to pest management with PSMs. Manipulation of genes involved in PSM biosynthesis pathways can also be helpful in combating aphid populations. Different enzymes are also involved in secondary metabolite biosynthesis, as well as gene expression in response to herbivore attacks. However, the use of PSMs to develop biopesticides has emphasized IPM strategies. Future research should leverage omics technologies and genetic engineering to develop herbivore-resistant varieties through the augmentation of secondary metabolites. In developing nations, farmers are not well-educated and motivated to use these approaches for sustainable agriculture. Farmers choose cost-effective synthetic pesticides for rapid outcomes instead of using eco-friendly biochemicals. Furthermore, there is a big study gap in understanding the entire mechanism of PSM application at the molecular or genetic level for aphids and other pest management. Investigation must be conducted in manipulating these secondary metabolites as biopesticides to manage aphids and save the environment from harmful pesticides.

## 7. Conclusions

This review describes the importance and application of PSMs, which have the potential to control the aphid population in agricultural crops and attract the natural counterparts of aphids, emphasizing safe and sustainable agriculture practices. In the future, utilizing these secondary metabolites at a large scale is important in combating the aphid population and overcoming the increasing insecticide resistance and environmental risk due to excessive use of synthetic pesticides. Furthermore, there is a big study gap in understanding the entire mechanism of PSM application at the molecular or genetic level for aphids and other pest management. Investigation must be conducted in manipulating these secondary metabolites as biopesticides to manage aphids and save the environment from harmful pesticides.

## Figures and Tables

**Table 1:** List of some major aphid species, host plants, distribution, and damage.

Aphid Species	Host Plants	Distribution	Damage	Reference
*Aphis gossypii*	Cotton, citrus, potato, cucurbits, tobacco, peppers, okra	Europe, Asia, USA, Australia, Africa	Shrinked and curled leaves, buds malformation, stunt growth, honeydew excretion, fungal growth, red leaf disease, cotton leaf curl virus.	[22]
*Acyrthosiphon gossypii*	Cotton, legumes	China, Ukraine, Central Asia, Europe	Damage seedling, shoot color fade, fruit drop, curling of leaves.	[23]
*Lipaphis erysimi pseudobrassicae*	Mustard, turnip, and cruciferous crops	Asia, Africa including South Africa, Benin, Sudan, Egypt, Mali	Honeydew secretion inhibits plant growth of sooty mold. Vector 13 different viruses, including Turnip mosaic, Radish mosaic, Cauliflower mosaic, and Beet mosaic virus.	[24,25]
*Myzus persicae*	Peach, potato	China, Australia, Asia, Europe	Sucks plant sap, necrosis, and chlorosis spots on fruits, reduces the market value of fruits, and transmits persistent and non-persistent viruses.	[26]
* Sitobion avenae *	Wheat	China, Asia, West Africa, Europe	Reduce seed weight, panicle, and size, annually 40% yield loss in China.	[27]
*Acyrthosiphon pisum*	Pea	USA, Northwest China,	Retarded growth, curling of leaves, wilting, transmit 30 different viruses including red clover vein mosaic, pea streak, and yellow mosaic virus, USD 60 million and 10–30% losses in China.	[28]
*Rhopalosiphum maidis*	Maize, barley, sorghum, wheat, oat	China, America, Latin America, Europe, New Zealand, Egypt, Canada, North Africa, Australia, India	Suck phloem sap, curling, yellowing, and drying of leaves, transmit maize dwarf mosaic and barley yellow dwarf virus, yield losses from 17.1–100% in India.	[29,30]
*Rhopalosiphum rufiabdominalis*	Rice, grasses, tomato, wheat	USA, Asia	Damage roots, develop defensive white wax to absorb extra moisture.	[31]
*Aphis glycines*	Soybean	Asia, USA, Latin America	Secrete honeydew, which promotes fungal growth reduction in photosynthetic activity, transmits deadly plant viruses, including soybean mosaic and alfalfa mosaic virus.	[32]

**Table 2:** Important PSMs with category, plant source, and defensive functions against aphid species.

PSM	Category of PSM	Insect Species	Plant Source	Defensive Function	Reference
**Terpenoids**					
Cucurbitacin B	Triterpenoid	*Aphis gossypii*	*Cucurbitaceae* family	Toxic to melon aphids and mites, Antifeedant	[19]
Farnesol	Sesquiterpenoid	*Myzus persicae*, *Macrosiphum rosae*, *Acyrthosiphon pisum*	Chamomile, lemongrass, tuberose	Pesticidal, Antimicrobial, Flavoring	[64,66,88,89]
*d*-Limonene	Monoterpenoid	*Aphis gossypii*, *Myzus persicae*, *Aphis citricola*, *Aphis craccivora*	Citrus, oranges	Aphicidal, Antioxidant	[62,90,91]
Saponins	Glycosides (Terpenoid)	*Acyrthosiphon pisum*	Oats, legumes	Antifeedant	[92,93]
Geraniol	Monoterpenoid	*Acyrthosiphon pisum*	Rose oil, lemongrass	Aphid repellent, Attract ladybug	[26]
Jasmone	Sesquiterpenoid	*Myzus persicae*, *Macrosiphum euphorbiae*	*Jasminum* spp., *Camellia sinensis*	Aphid repellent	[64,94]
Limonene, menthone, γ-terpinene, β-pinene, linalool, α-pinene	Terpenoids	*Macrosiphum roseiformis*, *Aphis citricola*, *Acyrthosiphon pisum*	Lemon/citrus extract	Aphicidal	[88,90,95]
Kaurene, ent-Kaurane, Clerodane	Diterpenes	*Aphis gossypii*	*Lamiaceae*	Aphicidal, Antimicrobial	[96]
Isomenthone, menthol, neomenthol	Monoterpenes	*Aphis gossypii*, *Myzus persicae*	*Mentha piperita*, *Mentha arvensis*	Aphicidal, Low fecundity	[97,98]
**Phenolics**					
Epigallocatechin Gallate	Flavonoid	*Aphis gossypii*	Cotton, green tea	Antioxidant, Antimicrobial, Antifeedant	[19,99]
Tannic acid	Tannin	*Aphis gossypii*, *Myzus persicae*, *Acyrthosiphon pisum*	*Quercus* and *Rhus* spp.	Antioxidant, Antifeedant, Antiviral	[100,101,102,103]
Pyrogallol, Gallic Acid, Ellagic Acid	Phenolics	*Schizaphis graminum*, *Acyrthosiphon pisum*, *Myzus persicae*, *Sitobion avenae*	*Oak* spp.	*Aphicidal*, *Antimicrobial*, *Anticancer*	[70,104]
Phloridzin	Flavonoid Glycoside	*Acyrthosiphon pisum*, *Eriosoma lanigerum*	*Malus* spp., *Rosaceae*	Antioxidant, Antifeedant	[105,106]
Daidzein, genistein, kaempferol, pigenin	Flavonoids	*Acyrthosiphon pisum*, *Aphis glycines*	Citrus, soybeans *Camellia sinensis*, *Trifolium pratense*	Antifeedant	[80,107,108,109,110,111]
Quercetin, luteolin, rutin	Flavonoids	*Macrosiphum rosae*, *Aphis spiraecola*	Berries, onions, citrus	Aphicidal, Antioxidant	[89,112,113,114,115]
Naringin, rutin hydrate	Flavonoids	*Eriosoma lanigerum*, *Myzus persicae*, *Aphis spiraecola*	Citrus, asparagus	Aphicidal, Antifeedant	[89,114,115,116,117]
Catechin	Flavonoid	*Macrosiphum rosae*, *Aphis spiraecola*	*Camellia sinensis* Berries	Aphid deterrence	[115,118]
Coumarin	Phenolic	*Myzus persicae*, *Aphis spiraecola*	*Dipteryx odorata*, *Cinnamomum*, *Melilotus* spp.	Toxic to aphids, Flavor enhancer	[115,119]
Xanthotoxin	Coumarin (Flavonoid)	*Melanaphis sacchari Zehntner*	*Ficus carica*, *Ficus petiolaris*	Aphicidal, Insecticidal	[120]
Condensed tannin	Proanthocyanidins	*Chaitophorus tremulae*, *Acyrthosiphon pisum*	*Populus tremula*	Antifeedant	[78,121]
**S-Containing PSMs**					
Camalexin	Phytoalexin	*Myzus persicae*, *Diuraphis noxia*	*Arabidopsis thaliana*	Plant defensive, Antifeedant	[122,123]
Glucosinolates	S-containing	*Myzus persicae*	*Arabidopsis thaliana*	Aphid repellent, Antifeedant	[75,124]
Propyl propane thiosulfonate, propyl propane thiosulfinate	S-containing	*Aphis gossypii*	*Allium cepa*	Antimicrobial, Aphid repellent	[125]
**N-containing PSMs**					
Capsaicin	Alkaloid	*Myzus persicae*, *Aphis gossypii*, *Aphis cytisorum*	*Capsicum* spp.	Antimicrobial, Pesticidal	[126,127,128,129,130]
Cytisine, sophoridine, aloperine, matrine, sophocarpine, oxymatrine	Alkaloids	Aphis craccivora, *Myzus persicae*, Sitobion avenae	*Sophora alopecuroides*	Aphicidal, Antioxidant	[84,131,132,133]
Amygdalin, prunasin	Cyanogenic glycosides	*Rhopalosiphm padi*	*Prunus* leaves	Antifeedant	[105,106,134,135]

## Data Availability

All data are contained within the article.
